# Fractional CO_2_ Laser Treatment of Anetoderma: Clinical Outcomes in a Sequentially Documented Case

**DOI:** 10.1111/jocd.71062

**Published:** 2026-07-20

**Authors:** Cherifa Nechat, Lidia M. Poppe

**Affiliations:** ^1^ Hautarztpraxis Dr. Poppe & Kollegen Bad Kissingen Germany

## 1 Introduction

Anetoderma is a rare dermatosis characterized by localized loss of dermal elastic tissue, resulting in soft, atrophic skin lesions [1]. These lesions are often round to oval, sharply demarcated, and can appear herniated or wrinkled [2]. Histological findings typically confirm the loss of elastic fibers in the mid‐dermis, with direct immunofluorescence tests showing no immune complex deposits [3]. The condition may arise without clear cause (primary anetoderma) or follow inflammatory or traumatic skin events (secondary) [4]. Prevalence is low and its actual incidence is unknown; it more commonly affects women and usually presents between ages 15 and 25 [5].

Despite its benign nature, anetoderma presents a significant cosmetic concern and lacks an effective standard treatment. Conventional therapies (including surgical removal, intralesional triamcinolone injections, and systemic therapies such as aspirin, dapsone, phenytoin, and hydroxychloroquine) offer limited improvement. More recent reports suggest that fractional CO_2_ laser therapy (ablative 10 600 nm) may represent a promising therapeutic alternative, showing encouraging clinical improvement in selected cases. This method creates controlled micro‐injuries in the skin and has been associated with improvement in the clinical appearance of several atrophic skin conditions. Evidence regarding its effectiveness in anetoderma remains limited [6].

This report presents a case of histologically confirmed anetoderma treated with a fractional CO_2_ laser (Exelo/Alma Lasers) over multiple sessions. It critically explores treatment decisions, parameters, objective assessments, and observed outcomes to assess whether targeted laser therapy can achieve measurable clinical improvement in a condition otherwise considered therapeutically challenging.

## 2 Case Presentation

A 40‐year‐old female patient of South American origin presented with multiple dermatological diagnoses. Primary concerns included biopsy‐confirmed anetoderma (L90.8) involving a large area along the back and chin with skin‐colored, sharply demarcated, round to oval lesions ranging from 0.2 to 1.0 cm. The lesions featured wrinkled, thinned skin, were partially sunken, and in some areas, subcutaneous fat herniation was visible. These changes were asymptomatic and often incidental. Hypopigmentation (L81.9) and scar tissue (L90.5) were also observed, with elastotic plaques present on the back and neck. Rosacea (L71.8), affecting the cheeks, nose, and chin, was considered a secondary diagnosis and was managed with doxycycline 40 mg orally once daily, in combination with alternating topical metronidazole cream and topical ivermectin. Attention‐deficit/hyperactivity disorder (ADHD), diagnosed in 2014, remained well controlled. Additional long‐term medication included oral hydroxychloroquine and l‐thyroxine 100 μg daily.

### 2.1 Laboratory and Diagnostic Findings

A history of elevated LDL and a positive ANA at 1:160 were documented. Serologies for hepatitis, HIV, and syphilis were negative. A repeat biopsy confirmed anetoderma. Direct immunofluorescence was negative. Family history included a sister with dermatosclerosis.

### 2.2 Therapeutic Approach

The initial consultation focused on the use of fractional CO_2_ laser (FRXL) therapy over fully ablative options, given the skin's texture and risk profile. FRXL was selected to treat the lower back and submandibular region. A total of five sessions were conducted over two years, using consistent settings with the CO_2_ laser device. Details of the therapeutic interventions are summarized in Table 1, and the laser treatment parameters are summarized in Table 2.

**TABLE 1:** Therapeutic interventions.

Session	Timing	Procedure	Settings	Areas treated	Post‐procedure care
Initial consultation	May, 2022	Discussion of treatment options	—	—	—
Trial session	September, 2022	Test treatment on left back	Topical anesthesia Ablative CO_2_: 10 mJ/3 ms Fractional CO_2_ (FRXL CO_2_): 30 mJ/2 ms (paintbrush mode)	Two spots on left back	Cooling + Fucidin + betamethasone ointment + biocellulose overlay
Session 1	May 2023	FRXL CO_2_	Topical anesthesia 30 J/cm^2^, 2 ms, 150 pts/cm^2^, 1 MHz, paintbrush mode	Back and submental region	Cooling + Fucidin + betamethasone ointment; panthenol cream; sun protection × 4 weeks
Session 2	Juli, 2023	FRXL CO_2_	Same as session 1	Same areas	Same post‐care
Session 3	August, 2023	FRXL CO_2_	Unchanged	Same areas	Same post‐care
Session 4	September, 2023	FRXL CO_2_	Unchanged	Same areas	Same post‐care
Session 5 (Final)	May 2024	FRXL CO_2_	Unchanged	Same areas	Same post‐care

**TABLE 2:** Summary of laser treatment parameters.

Parameter	Details
Device	Fractional CO_2_ Laser (Exelo, Alma Lasers)
Energy density	30 J/cm^2^
Pulse duration	2 ms
Dot density	150 points/cm^2^
Frequency	1 MHz
Delivery mode	Paintbrush mode
Anesthesia	Topical
Treatment sites	Back and submental region
Post‐treatment care	Cooling, biocellulose mask, topical antibiotic/steroid combination initially, panthenol gel, strict sun protection for 4 weeks

Rationale for treatment schedule: an initial test session (fractional vs. ablative) was performed to evaluate safety and tolerability. Fractional CO_2_ sessions were scheduled roughly every 6–10 weeks to allow complete re‐epithelialization and dermal remodeling between treatments, consistent with protocols for scar remodeling and fractional ablative resurfacing. The decision to continue up to five sessions over approximately two years was based on progressive clinical response and patient preference to continue treatment until improvement plateaued; intervals were extended as needed for healing and to monitor cumulative effect.

Clinical photography was performed at each visit using the same camera model under standardized conditions when possible. Minor variations in patient positioning, angle, and ambient lighting may have occurred due to the clinical setting. Images were acquired in a consistent environment to minimize variability. Pre‐treatment images were obtained at baseline (May 2022), and post‐treatment images were captured immediately after the initial treatment session (September 2022). Additional interim photographs were taken after sessions 2 and 3, while final follow‐up images were obtained after session 5 (May 2024).

## 3 Objective Assessments

Photographs were independently evaluated by two blinded dermatologists using a 5‐point global aesthetic improvement scale (GAIS: 0 = worse, 1 = no change, 2 = mild, 3 = moderate, 4 = marked). Patient‐reported outcome included a 10‐point satisfaction scale. Inter‐rater agreement for GAIS was calculated with Cohen's kappa. High‐frequency ultrasound was not available in this case; future studies should include ultrasound or cutometer testing for elasticity and dermal thickness assessment.

- Initial consultation: discussion of treatment options, including ablative and fractional CO_2_ laser.
- Trial session: test treatment on left back comparing ablative CO_2_ (10 mJ/3 ms) and fractional CO_2_ (30 mJ/2 ms, paintbrush mode). Topical anesthesia was used in two areas. Post‐care included Fucidin and betamethasone ointment twice daily for five days and a biocellulose overlay. FRXL was chosen due to lower invasiveness.
- Session 1: Full session with FRXL CO_2_. Energy density 30 J/cm^2^, pulse duration 2 ms, density 150 pts/cm^2^, frequency 1 MHz in paintbrush mode. Treated: back and submental region. Topical anesthesia applied. Post‐laser care: cooling, Fucidin and betamethasone ointment followed by panthenol cream. Sun protection for four weeks.
- Sessions 2–5 were performed with unchanged parameters and consistent post‐care; clinical response was monitored throughout, device function was rechecked when applicable, treatment zones remained unchanged, and outcomes were documented at the final session.

## 4 Findings Across Sessions

Progressive improvement was noted after each laser treatment. The wrinkled texture and sunken appearance of the anetodermic lesions on the back and submental areas showed visible tightening and a more uniform skin tone.

The submandibular chin region showed a clinically firmer appearance and less visible skin laxity. Lesion margins became less distinct, and the affected areas appeared cosmetically improved on serial photographic assessment. Patient‐reported outcomes were positive, with no procedural complications reported.

Objective outcomes: Blinded GAIS scoring by two dermatologists rated the final improvement as moderate‐to‐marked (median GAIS = 3). High‐frequency ultrasound was not available; no histologic post‐treatment sampling was performed.

**FIGURE 1:**
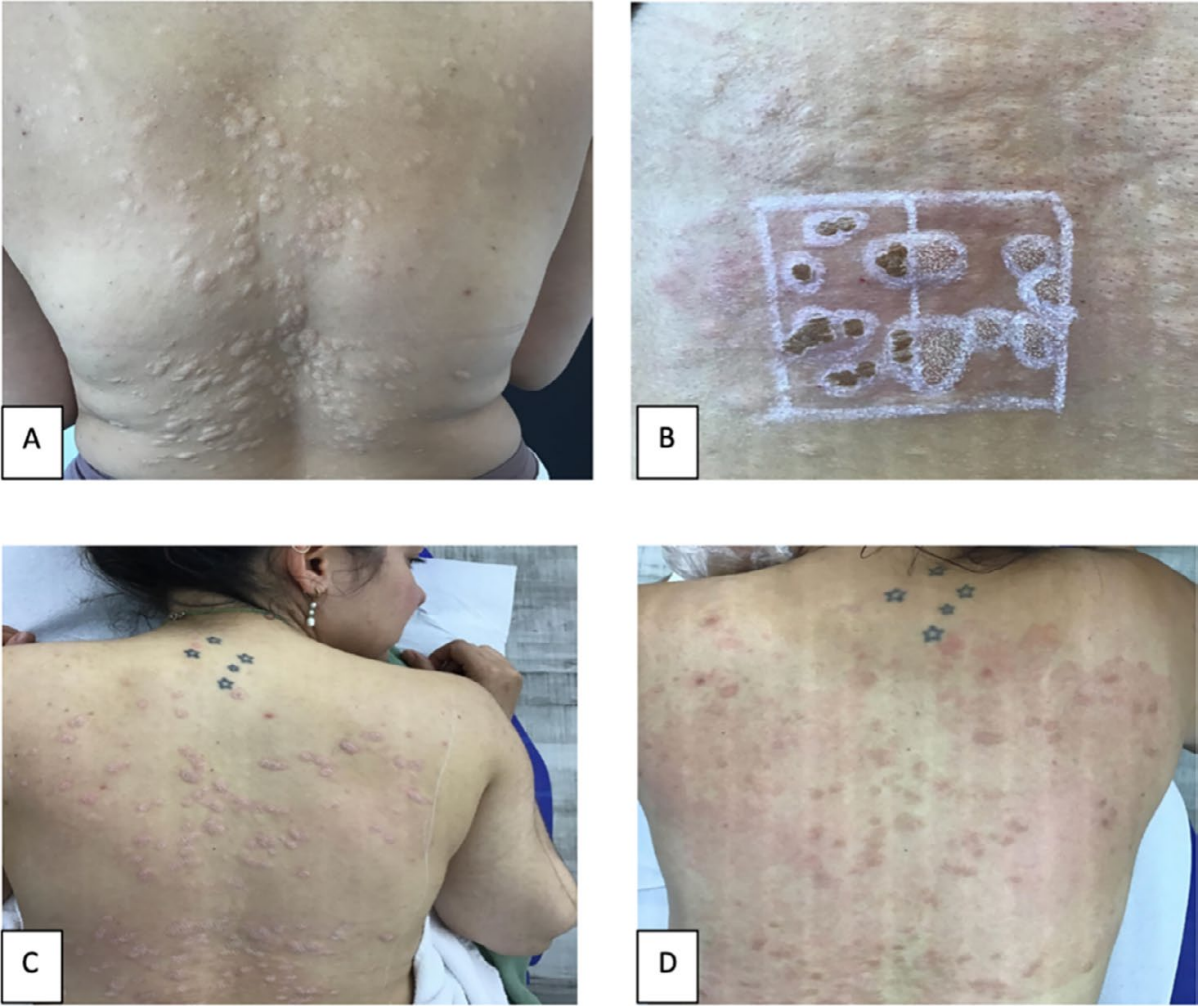
Standardized clinical images showing progression. (A) Baseline before any intervention (May, 2022). (B) Test session baseline showing fractional (right) and ablative (left) treatment (September, 2022). (C) Captured immediately post‐laser session, showing typical post‐procedural erythema (September, 2023). (D) Post‐treatment after full‐back fractional CO_2_ application (May, 2023).

**FIGURE 2:**
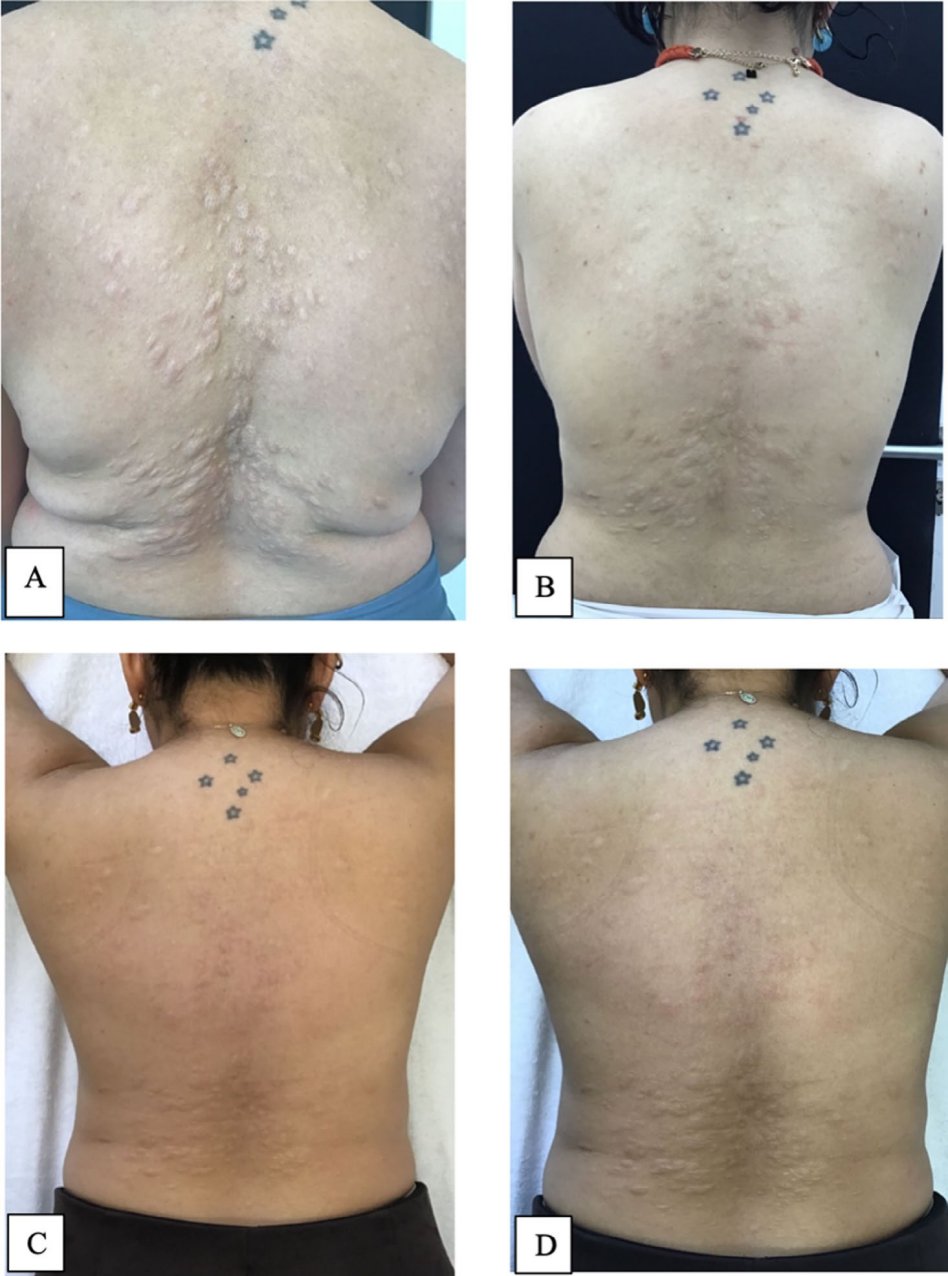
Standardized clinical images demonstrating treatment outcomes following fractional CO_2_ laser sessions. (A) Baseline Images from September 2022. (B) Images from May 2024, showing improvement in anetodermic lesions, including reduction in lesion protrusion, improved skin texture, and flattening of the affected areas. (C, D) Images from February 2026. The wrinkled and depressed anetodermic lesions on the back demonstrated visible skin tightening and a more uniform skin tone. Lesion margins became less distinct, and the previously thinned epidermis regained partial structural integrity.

Images documented the evolving clinical picture. Initial pre‐treatment photos from the trial session provided a baseline. Intermediate images taken after the second and third sessions showed visible tightening and retraction of atrophic skin areas. Final images taken after the fifth session confirmed improvement in both texture and contour (Figures 1 and 2).

## 5 Discussion

This case showed progressive improvement of biopsy‐confirmed anetoderma following repeated fractional CO_2_ laser (FRXL) therapy. The patient's lesions, which had remained unchanged for years despite no history of trauma, infection, or inflammation, responded to a structured FRXL protocol after standard treatments failed to provide satisfactory cosmetic improvement. Fractional CO_2_ was chosen over fully ablative laser to balance efficacy with safety, as fully ablative approaches pose higher scarring risk in atrophic skin.

Proposed mechanisms and interpretation: the clinical improvement observed in this case may be related to tissue responses previously described after fractional CO_2_ laser treatment. However, because no histologic, molecular, or biomechanical assessments were performed, the biological mechanisms underlying the observed changes cannot be determined from this report.

Whether elastic fiber regeneration or other structural changes occurred remains unknown. These proposed mechanisms should be considered hypotheses requiring histologic or biomechanical confirmation in future studies.

Prior reports and context: Published data on laser treatment for anetoderma are limited to case reports and small series. Cho et al. described successful use of an ablative 10 600‐nm fractional CO_2_ laser to improve anetoderma secondary to Stevens–Johnson syndrome [6]. Other literature on laser use in atrophic dermal conditions supports induction of neocollagenesis after fractional CO_2_ but lacks series specifically on primary anetoderma. No randomized or controlled studies exist. This paucity of data underscores the preliminary nature of our observations and the need for systematic studies comparing fractional versus ablative approaches, objective biomechanical measures, and histologic correlation.

### 5.1 Limitations

This is a single‐patient case report with no histologic post‐treatment confirmation, limited objective baseline measures (no pre‐ and post‐treatment biopsy or ultrasound), and potential bias from retrospective planimetry and photographic assessment. The absence of long‐term stability data and the single‐case design limit generalizability and preclude causal assertions. Future studies should include larger cohorts, prospective standardized objective measurements (e.g., cutometer elasticity testing, high‐frequency ultrasound, histology), blinded assessments, and controlled designs.

### 5.2 Safety and Tolerability

The patient tolerated treatment well, reporting only transient erythema and mild crusting. The use of biocellulose masque and topical antibiotics supported epithelial recovery. No secondary infections, hypertrophic scarring, or dyspigmentation were noted. This safety profile is notable given darker phototypes (Fitzpatrick IV) and structurally fragile skin, though larger series are required to better define safety.

## 6 Conclusion

Fractional CO_2_ laser therapy may be a promising, minimally invasive option to improve the clinical appearance of localized anetoderma when conventional therapies fail. However, evidence remains preliminary. Observed clinical improvements in this single case warrant controlled studies incorporating objective clinical, biomechanical, and histologic assessments to better define treatment outcomes and optimal treatment parameters.

## Funding

The authors have nothing to report.

## Ethics Statement

All research was conducted with integrity and in accordance with ethical standards.

## Consent

Written informed consent was obtained for treatment and for publication of anonymized clinical images.

## Conflicts of Interest

The authors declare no conflicts of interest.

## Data Availability

The data that support the findings of this study are openly available in Pubmed [1, 2, 3, 4, 5, 6].
